# Microstructural Evolutions of 2N Grade Pure Al and 4N Grade High-Purity Al during Friction Stir Welding

**DOI:** 10.3390/ma14133606

**Published:** 2021-06-28

**Authors:** Tomoya Nagira, Xiaochao Liu, Kohasaku Ushioda, Hidetoshi Fujii

**Affiliations:** 1 Joining and Welding Research Institute, Osaka University, 11-1 Mihogaoka, Ibaraki, Osaka 567-0047, Japan; kohsaku.ushioda@26iron56.sakura.ne.jp (K.U.); fujii@jwri.osaka-u.ac.jp (H.F.); 2 National Institute for Materials Science, 1-2-1 Sengen, Tsukuba, Ibaraki 305-0047, Japan; 3 School of Mechanical Engineering, Southeast University, Nanjing 211189, China; xcliu1990@seu.edu.cn

**Keywords:** friction stir welding, aluminum, microstructural evolution, dynamic recrystallization

## Abstract

The grain refinement mechanisms along the material flow path in pure and high-purity Al were examined, using the marker insert and tool stop action methods, during the rapid cooling friction stir welding using liquid CO_2_. In pure Al subjected to a low welding temperature of 0.56*T_m_* (*T_m_*: melting point), the resultant microstructure consisted of a mixture of equiaxed and elongated grains, including the subgrains. Discontinuous dynamic recrystallization (DDRX), continuous dynamic recrystallization (CDRX), and geometric dynamic recrystallization are the potential mechanisms of grain refinement. Increasing the welding temperature and Al purity encouraged dynamic recovery, including dislocation annihilation and rearrangement into subgrains, leading to the acceleration of CDRX and inhibition of DDRX. Both C- and B/$\hat{B}$-type shear textures were developed in microstructures consisting of equiaxed and elongated grains. In addition, DDRX via high-angle boundary bulging resulted in the development of the 45° rotated cube texture. The B/$\hat{B}$ shear texture was strengthened for the fine microstructure, where equiaxed recrystallized grains were fully developed through CDRX. In these cases, the texture is closely related to grain structure development.

## 1. Introduction 

Friction stir welding (FSW) has been recognized as a promising candidate for a solid-state joining technique, which offers several potential advantages including the avoidance of both crack formation and high distortion [1,2,3,4]. The FSW of Al alloys has several industrial applications, such as automotive, aerospace, and train [5,6]. Numerous studies have investigated the microstructural features of Al alloys, which are a result of complex plastic deformation phenomena at high temperatures, including recovery, recrystallization, and grain growth during FSW [6,7,8,9,10,11,12,13,14,15,16]. Initially, the microstructural change from base materials to the stir zone was examined for the FSWed joint on the transverse and horizontal cross sections [7,8,9,10,11]. To obtain a better understanding of the microstructural evolution during FSW, the stop action method, where the FSW machine is emergently stopped and the tool is immediately lifted from the work piece, followed by rapid cooling using a mixture of methanol and dry ice or water, was adopted by Fonda et al. [12], Prangnell et al. [13], Su et al. [14], and Suhuddin et al. [15]. This method facilitated the preservation of the developing microstructure around the exit hole. Su et al. proposed multiple grain refinement mechanisms, including discontinuous dynamic recrystallization (DDRX), dislocation introduction, and continuous dynamic recrystallization (CDRX) [14]. Suhuddin et al. demonstrated that geometrical effects as a result of strain, as well as discontinuous and continuous recrystallization, induced grain refinement ahead of the tool pin [15]. However, grain structure development along the material flow path during FSW is yet to be elucidated. Furthermore, the marker insert method, in which a thin marker material is inserted at the joint line, has been combined with the stop action method to examine the complex material flow during FSW [16]. The material flow path around the tool can be visualized by the marker insert method. Based on these techniques, Liu et al. simultaneously combined the marker insert and stop action methods during more rapid cooling FSW using liquid CO_2_ [17] in our previous studies [18,19,20,21]. Herein, we examined the microstructural evolution along the material flow path in FCC metals, exhibiting different stacking fault energies (SFEs), such as Al (166 mJ·m^−2^) [18], Cu (78 mJ·m^−2^) [20], and Cu-30Zn (20 mJ·m^−2^) [19], and revealed that the dominant grain refinement mechanism during FSW changed from CDRX in the Al to DDRX in Cu-30Zn with decreasing the SFE.

Several other factors beside the SFE affect the microstructural evolution during FSW. The welding temperature [22,23,24] and mobility of the grain boundaries [24] also influence the microstructure through recovery, recrystallization, and grain growth. In our previous studies [22,23,24], the effect of the welding temperature on the recrystallization behavior of high-purity Ag with a low SFE (22 mJ·m^−2^) was examined using the stop action and marker insert methods during rapid cooling FSW. The microstructure was refined by the frequent annealing twinning, and no dynamic recrystallization occurred at 0.59*T_m_* (*T_m_*: melting point). However, a decrease in the welding temperature to 0.36*T_m_* resulted in the accumulation of dislocations along the grain boundaries, owing to an inhibition of dynamic recovery and decreased mobility of grain boundary, leading to the occurrence of DDRX. Our previous studies also revealed that DDRX was accelerated through the addition of a small amount of Sn (0.75 wt%) to pure Ag [24]. The addition of a solute imparted the same effect on the recrystallization behavior as a decrease in the welding temperature.

In the case of Al with a high SFE, the effect of the welding temperature on the microstructural evolution during FSW between the temperature range of 0.45*T_m_* and 0.77*T_m_* was examined by Mironov et al. [11]. The grain refinement occurred through CDRX irrespective of the welding temperature, and an increase in the welding temperature resulted in a transformation from lamellar to equiaxed-type grains. It should be noted that the stop action, rapid cooling, and marker insert methods were not used in this test. The microstructure can be influenced by the annealing during the cooling stage of the FSW. The microstructural evolution along the material flow path during FSW is not yet fully understood.

In this study, the microstructural evolution of pure Al during FSW along the material flow path was examined through a simultaneous combination of the marker insert and tool stop action methods while rapidly cooling using liquid CO_2_. The effects of the welding temperature and Al purity on the grain refinement mechanism and texture evolution, with respect to dynamic recrystallization during FSW, were examined.

## 2. Materials and Methods

**Figure 1:**
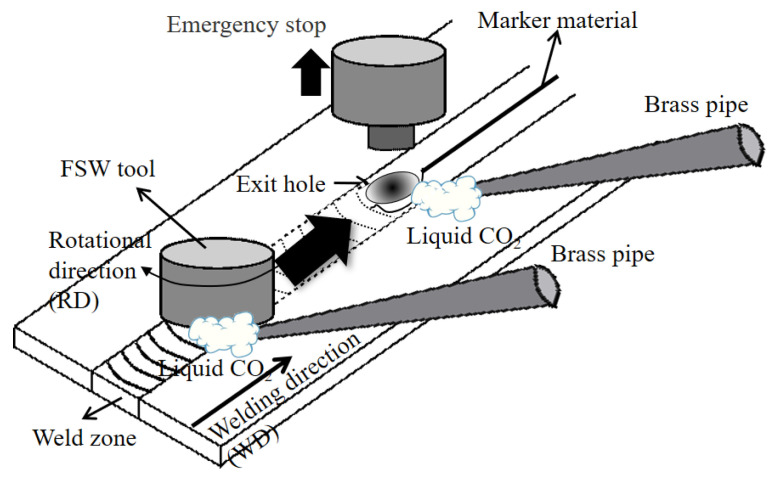
Schematic of the experimental procedure.

The marker insert and tool stop action methods during rapid cooling FSW are outlined schematically in Figure 1. An Al alloy (AA6061) foil of 0.5 mm thickness was inserted into the abutting surface of the two workpieces as a marker material to trace the material flow path. Liquid CO_2_ was jetted onto the fresh weld surface through brass pipes during FSW. When the tool went forward 40 mm along the abutting surface, where the marker material was inserted, the tool was immediately lifted from the work piece by activating the emergency stop of the FSW machine during the application of the liquid CO_2_. Note that the transient microstructures under the tool shoulder and around the exit hole were completely quenched. Therefore, grain growth through the annealing during the cooling stage of the FSW can be sufficiently suppressed [17,18].

Commercial pure 2N grade Al (cold-rolled AA1050) and high-purity Al (4N grade) were used as the base materials. Table 1 shows the chemical compositions of pure and high-purity Al. The dimensions of both plates were 100 mm in length, 100 mm in width, and 3 mm in thickness. The FSW tool consists of a planar shoulder with a diameter of 12 mm and a smooth cylindrical probe with a diameter and length of 4.0 mm and 2.8 mm, respectively. The tilt angle of the tool was 3° with respect to the normal direction. The FSW machine was operated in the displacement control mode with a constant penetration depth of 2.8 mm. For pure Al, butt welding was conducted under both low and high heat input conditions, where the tool rotational speeds were 800 and 1500 rev/min, respectively. In contrast, for high-purity Al, the tool rotational speed was 1500 rev/min. The tool traveling speed was set at 100 mm/min for both specimens. The specimens, including the exit hole, were fabricated using a wire-cut electric discharge machine and polished to a depth of 0.5 mm from the surface, where the shoulder-affected zone is located [25]. For optical microscopy observations and electron backscattering diffraction (EBSD) measurements, the polished specimen was electrically etched in a solution of HClO_4_ : C_2_H_5_OH = 1 : 4 at ~0 °C (ice bath) with an applied potential of 20 V. The EBSD measurements were carried out at 15 kV along the flow path of the marker material on the Al. The EBSD maps were obtained with a step size of 0.15–0.30 μm. The grain size was calculated as a circle equivalent diameter of each grain in the EBSD map. The low-angle boundaries (LAB) and high-angle boundaries (HAB) were defined by misorientation angles (*θ*) of 2° < *θ* < 15° and *θ* > 15°, respectively. To characterize the grain structure development across the transition from the unwelded to the welded zone, an EBSD measurement position in a direction parallel to the welding direction was defined as a function of the distance, x mm, from the center of the exit hole. The peak welding temperature during the FSW was measured using a K-type thermocouple placed on the bottom surface of the plates at the centerline. Table 2 lists the welding conditions and final grain sizes for pure and high-purity Al. The peak welding temperatures for pure Al at tool rotational speeds of 800 and 1500 rev/min were 523 (=*T_800_*) and 629 K (=*T_1500_*), respectively. For high-purity Al, the peak welding temperature at a tool rotation speed of 1500 rev/min was 465 K (=*T_1500_*). It is noted that the temperature within the 4 mm diameter probe is almost equivalent to the peak temperature. In this study, the welding condition was defined as a ratio of peak temperature to melting point of 933 K [26]. Therefore, *T_800_*/*T_m_* and *T_1500_*/*T_m_* were 0.56 and 0.67 for pure Al, respectively. *T_1500_*/*T_m_* was 0.50 for high-purity Al.

## 3. Results and Discussion

### 3.1. Pure Al

**Figure 2:**
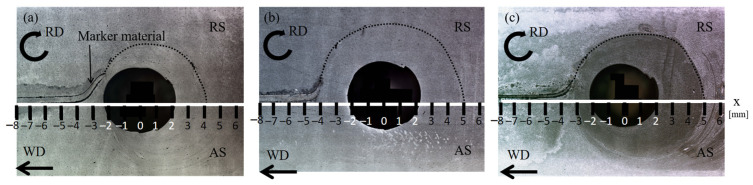
Optical micrographs of horizontal sections of FSW joints for pure Al at (**a**) 0.56*T_m_* and (**b**) 0.67*T_m_*; (**c**) high-purity Al at 0.50*T_m_*.

Figure 2a,b shows optical microscopy images of the horizontal section of the pure Al joints produced at the peak temperatures of 0.56*T_m_* and 0.67*T_m_*, respectively. The marker materials flowed around the exit hole toward the retreating side (RS), which corresponds to the shoulder-affected zone with a diameter of 12 mm [25]. In the range of −8.0 < x < −4.0, the marker material gradually became thinner for both specimens, then was remarkably reduced at x = −3.5 and −4 for pure Al at 0.56*T_m_* and 0.67*T_m_*, respectively. The dotted black lines indicate the distribution of the marker materials after a significant reduction in their thickness. The flow range in the stir zone for the pure Al joint at 0.67*T_m_* was wider than that at 0.56*T_m_* due to the reduced flow stress at increased temperatures. The features of the flow pattern were similar to those of the FCC metals with a low SFE in our previous studies [19,20,21,22,23,24].

#### 3.1.1. Microstructural Evolution at 0.56*T_m_*

**Figure 3:**
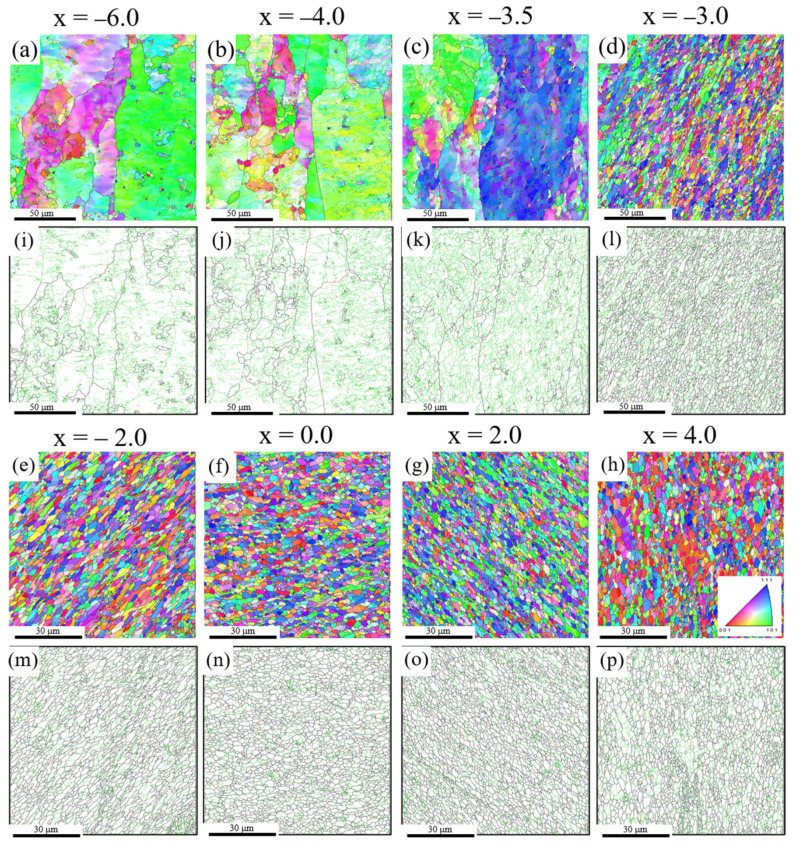
(**a**–**h**) IPF and (**i**–**p**) grain boundary maps for the pure Al joint at 0.56*T_m_* in the range of −6 < x < 4. Black and green lines denote HABs and LABs, respectively.

**Figure 4:**
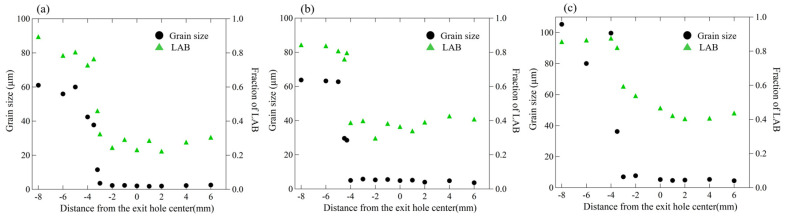
Average grain size and fraction of LABs for pure Al at (**a**) 0.56*T_m_* and (**b**) 0.67*T_m_*; (**c**) high-purity Al at 0.50*T_m_*.

**Figure 5:**
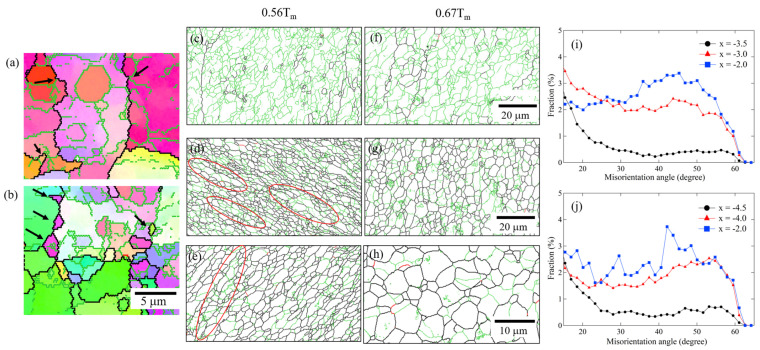
Magnified (**a**–**b**) IPF maps of Figure 3(**b**) at x = −4.0. Magnified grain boundaries maps at (**c**) x = 3.5, (**d**) x = −3.0, and (**e**) x = −2.0 at 0.56*T_m_*; and (**f**) x = 4.5, (**g**) x = −4.0, and (**h**) x = −2.0 at 0.67*T_m_*. Misorientation histograms of the HAB at (**i**) 0.56*T_m_* and (**j**) 0.67*T_m_*.

Figure 3a–p shows the inverse pole figure (IPF) and grain boundary maps at −6.0 < *x* < 4.0, respectively. Figure 4a shows the average grain size and fraction of LABs at −8.0 < *x* < 6.0. For the base material at x = −8.0, there were a significant number of LABs throughout the original large grains due to the slight deformation by cold rolling. The fraction of LABs and average grain size were 84% and 64 μm, respectively. In the range of −8.0 < *x* < −5.0, the average grain size and fraction of LABs remained almost unchanged. Although the strain increased at x = −4.0, the fraction of LABs still remined constant. In contrast, the average grain size reduced to 42 μm, owing to nucleation of several fine grains along the original grain boundaries. The magnified IPF maps at x = −4.0 are shown in Figure 5a,b. The bulging of HABs induced by the difference in the dislocation density between the grain boundaries frequently occurred, as indicated by the arrows in Figure 5a. As a result, fine recrystallized grains were formed along the grain boundaries, which corresponds to the conventional DDRX [27], as denoted by the arrows in Figure 5b. It has been reported that DDRX via HABs bulging usually occurred in FCC metals with a low SFE, such as Cu [20,23], Cu-30Zn [19,23], and Ag [22,24]. The low SFE is presumed to hinder the climb and cross slip of dislocations during deformation, leading to the increase in the dislocation density, which provides the driving force for DDRX. In contrast, the high SFE causes the dynamic restoration, including dislocation annihilation and rearrangement [27]. However, Mironov et al. reported that the HAB bulging was observed far from the stir zone in FSWed Al (AA1050) [11]. In this study, DDRX likely occurred due to the low temperature operation of FSW, despite the high SFE.

At x = −3.5. where the marker material began to deform significantly, a large number of fine subgrains with an average diameter of 3.5 μm were formed within the original large grains, as shown in Figure 3c,k and Figure 5c. This can be attributed to the progressive accumulation and rearrangement of dislocations induced by the dynamic recovery during plastic deformation, which is generally observed in FCC metals with a high SFE during hot deformation [27]. The fractions of misorientation angles (*θ* > 20°) were less than 1%, as shown in the misorientation histogram of HAB (Figure 5i). When the strain increased further at x = −3.0, where the marker material was severely deformed, a significant number of equiaxed grains, with an average diameter of 3.0 μm, were distributed, as shown in Figure 3d,l, and 5d. The fraction of LABs decreased significantly from 76% at x = −3.5 to 30% at x = −3.0, as shown in Figure 4a. In contrast, the fractions of misorientation angles (20° < *θ* < 55°) significantly increased by more than two times at x = −3.0, as shown in Figure 5i. This indicates that conventional CDRX occurred, in which LABs transform into HABs through the gradual increase in misorientation via the rotation of subgrains [8,18]. In addition, elongated grains with a high aspect ratio were frequently observed, as indicated by the red circles in Figure 5d. The elongated grains with the serrated HABs were divided by the LABs, and the width of the minor axis of the elongated grains was reduced to the dimensions of the subgrain. These are typical features of geometric dynamic recrystallization (GDRX) [28]. In GDRX, the elongated grains finally transform into equiaxed grains through the pinching-off of the subgrain boundaries [27].

At x = −2.0 and 0.0, where the welding temperature reached a maximum, the microstructure consisted of finer grains with an average diameter of 2 μm, as shown in Figure 3e,f,m,n. The fully recrystallized fine microstructure was formed by the repeated formation of subgrains inside the large grains, following the transformation from the LABs to HABs. During this process, the fractions of misorientation angles in the range of 35° to 55° increased, as shown in Figure 5i, and began to approach to a random distribution. In addition, elongated grains, including subgrains, were partially observed, as shown in the red circle of Figure 5e. This indicates that the microstructure was refined by the GDRX, as well as the CDRX, at the peak temperature. As the x-position tracked away from the exit hole in the range of 2.0 < x < 6.0, the average grain size (2 μm) and fraction of LABs (30%) remained almost unchanged, as shown in Figure 4a. The subgrains continued to be formed inside the grains, as shown in Figure 3o,p. The fine microstructure was likely maintained through a dynamic balance between grain growth by heating and grain refinement by CDRX and GDRX.

#### 3.1.2. Microstructural Evolution at 0.67*T_m_*

**Figure 6:**
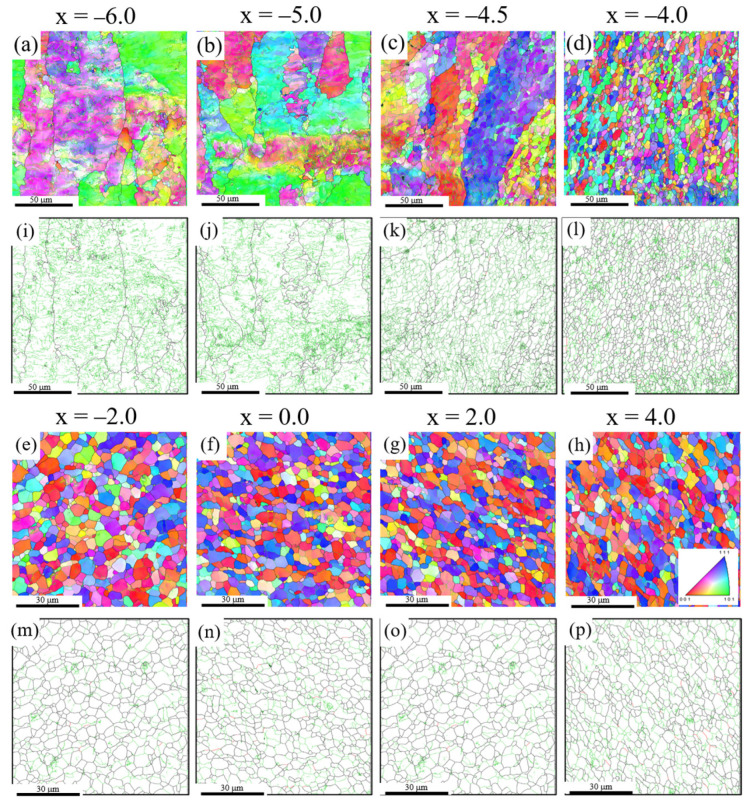
(**a**–**h**) IPF and (**i**–**p**) grain boundary maps for the pure Al joint at 0.67*T_m_* in the range of −6.0 < x < 4.0.

The microstructural evolution was examined at a higher welding temperature of 0.67*T_m_*. Figure 6a–p shows the IPF and grain boundary maps in the range of −6.0 < *x* < 4.0, respectively. Figure 4b shows the average grain size and fraction of LABs in the range of −8.0 < *x* < 6.0. In the range of −8.0 < *x* < −6.0, the average grain sizes (84%) and fraction of LABs (63 μm) were almost constant. Despite the strain increased at x = −5, DDRX induced through the bulging of HABs hardly occurred. An increase in the welding temperature resulted in an inhibition of DDRX, which is consistent with our previous studies regarding the welding temperature dependence on the microstructural development during FSW [22,23,24]. When the welding temperature is approximately 1.6 times higher than the recrystallization temperature, DDRX is completely suppressed owing to the promotion of dynamic recovery, including dislocation annihilation in FCC metals with a low SFE below 80 mJm^−2^ [23]. In this study, the ratios of the peak temperature to the recrystallization temperature for pure Al at 0.56*T_m_* and 0.67*T_m_* are 1.1 and 1.3, respectively. The increase in the temperature for high-SFE metals is more likely to induce significant dynamic recovery than in low SFE metals.

At x = −4.5 where the marker material began to deform, a significant number of subgrains with an average diameter of 4.0 μm were distributed within the original large grains, as shown in Figure 5f and Figure 6k. The more equiaxed and larger size subgrains were formed when compared to the grains of the pure Al at a low welding temperature of 0.56*T_m_* (Figure 5c). The fractions of misorientation angles (*θ* > 20°) were less than 1%, as shown in Figure 5j. As the strain increased further at x = −4.0, the microstructure consisted of only equiaxed fine grains with an average diameter of 5.0 μm, as shown in Figure 5g and Figure 6l. The fraction of LABs significantly decreased from 80% at x = −4.5 to 40% at x = −4.0, as shown in Figure 4b, whereas the fractions of misorientation angles (20° < *θ* < 60°) significantly increased, as shown in Figure 5j. This implies that CDRX occurred through the transformation of LABs to HABs. In general, more dynamic recovery occurs at higher temperatures, and the stored energy, which provides the driving force for recrystallization, is reduced [27], leading to an acceleration of CDRX.

In the range of −2.0 < x < 2.0, which corresponds to the peak temperature range, equiaxed recrystallized grains with an average diameter of 5 μm were uniformly distributed, as shown in Figure 6e–g. For the high-purity Al, the fraction of misorientation angles in the range of 40° to 50° increased significantly, reaching closer to the ideal random distribution [27] than that for pure Al at 0.56 *T_m_*, as shown in Figure 5i,j. This suggests that the transformation from subgrains to HABs is more likely to occur at higher temperatures, owing to increased grain boundary mobility. In addition, the grain size and morphology remained unchanged, even at the peak temperature (Figure 4b). LABs were frequently formed inside the grains, as shown in Figure 5h and the fraction of LABs was approximately 40%. Accordingly, the frequent occurrence of CDRX contributed to the inhibition of grain growth induced by the heating effect. In the range of 2.0 < x < 6.0, the grain size (4.7 μm) and the LABs fraction remained unchanged (Figure 4b). This can also be attributed to the dynamic balance between grain growth by the heating effect and grain refinement by CDRX.

### 3.2. High-Purity Al

The microstructural evolution of high-purity Al was examined at 0.50*T_m_*. An optical microscopy image of a horizontal section is shown in Figure 2c. Despite a tool rotational speed of 1500 rev/min, which was the same as that for the pure Al under the high heat input condition (0.67*T_m_*), the peak temperature of high-purity Al was similar to that under the low heat input condition in pure Al (800 rev/min, 0.56*T_m_*). In general, the heat is generated by plastic deformation, as well as friction effect during friction stir welding. Therefore, the heat generated by the plastic deformation varies with mechanical properties of materials. The tensile strength of 2N grade pure Al is approximately three times higher than that of 4N grade high-purity Al [29]. Thus, the heat generated by the mechanical working in the high-purity Al during FSW was lower than that generated in the pure Al. As shown in Figure 2b,c, the flow range of the high-purity Al was narrower than that of the pure Al under the high heat input condition (0.67*T_m_*), while the deformation behavior of the marker material was similar to that of pure Al at the lower welding temperature of 0.56*T_m_*.

**Figure 7:**
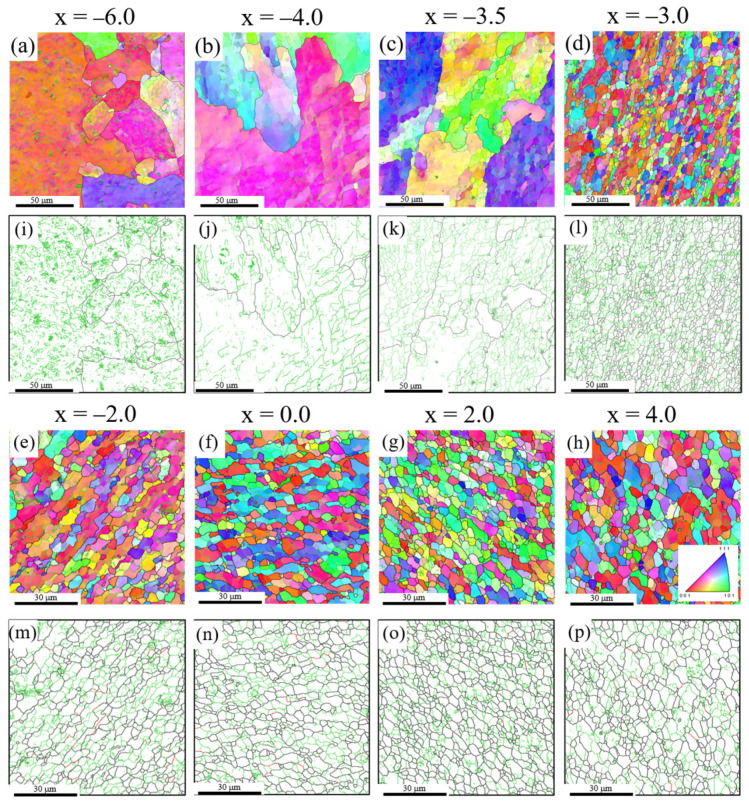
(**a**–**h**) IPF and (**i**–**p**) grain boundary maps for the high-purity Al joint at 0.50*T_m_* in the range of −6.0 < x < 4.0.

Figure 7a–p shows the IPF and grain boundary maps in the range of −6.0 < *x* < 4.0, respectively. Figure 4c shows the average grain size, and fraction of LABs in the range of −8.0 < *x* < 6.0. In the range of −8.0 < *x* < −4.0, the average grain size (86%) and fraction of LABs (90 μm) remained almost unchanged. At x = −4.0, where the marker material began to deform, no DDRX via HAB bulging occurred, as shown in Figure 7b,j. However, DDRX tended to occur more frequently in pure Al at a lower welding temperature of 0.56*T_m_*, as shown in Figure 5a,b. In our previous study, although no DDRX occurred during FSW of the 4N grade high-purity Ag, the addition of a small amount of Sn caused DDRX, owing to an increase in the dislocation density due to the suppression of dynamic recovery [24]. In contrast, it is reported that the high-purity Al enhances the grain boundaries mobility, leading to the occurrence of DDRX via HAB bulging [30]. This imparts the opposite effect of promoting DDRX. Accordingly, the effect of promoting dynamic recovery in high-purity Al likely exceeded the effect of increasing the grain boundary mobility, resulting in no occurrence of DDRX.

At x = −3.5, where the marker materials started to deform, a large number of subgrains with an average diameter of 4.5 μm were developed within the larger original grains, as shown in Figure 7c,k. As the strain increased further at x = −3.0, a large number of equiaxed fine grains with an average diameter of 4.7 μm were observed, as shown in Figure 7d,l. Elongated grains were hardly formed, and the fraction of LABs was significantly reduced to 40% (Figure 4c). Accordingly, fine grains were formed through CDRX via the transformation of LABs into HABs. As in the case of the pure Al, the fraction of misorientation angles (*θ* > 20°) increased significantly. At x = −2.0, where the welding temperature reached the peak one, the average grain size of high-purity Al was 4.8 μm, which was approximately equal to that of pure Al at a higher welding temperature of 0.67*T_m_*, as shown in Table 2. The addition of solute elements generally decreases the mobility of grain boundaries, owing to the solute drag effect [27]. Therefore, an increase in the purity facilitated grain growth as well as the acceleration of CDRX owing to the enhancement of the dynamic recovery and increased mobility of the grain boundaries. Moreover, an increase in the purity decreases the recrystallization temperature [31]. The recrystallization temperature of 2N grade pure Al is approximately 473 K. In contrast, the recrystallization temperature of 4N grade high-purity Al is within a range of 333–443 K. Therefore, it is likely that the dynamic recrystallization for the 4N grade high-purity Al occurs easily at a lower welding temperature than that for the 2N grade pure Al. The relationship between the recrystallization temperature, which depends on the purity, and dynamic recrystallization during FSW remains to be investigated in a future study. In the range of −2.0 < x < 6.0, the average grain size (4.8 μm) and the LABs fraction (40%) remained unchanged, as shown in Figure 4c. As in the case of pure Al, grain growth by the heating effect was dynamically balanced with grain refinement by CDRX.

### 3.3. Texture Development

**Figure 8:**
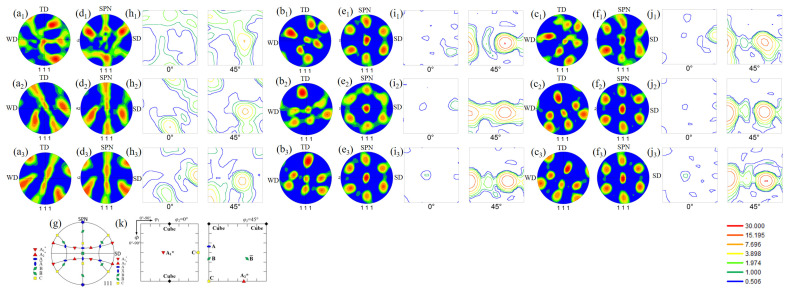
Original (111) pole figures at x = −3.0, 0.0, and 2.0 for pure Al at (**a_1_**–**a_3_**) 0.56*T_m_* and (**b_1_**–**b_3_**) 0.67*T_m_*; (**c_1_**–**c_3_**) high-purity Al at 0.50*T_m_*. (**d_1_**–**d_3_**), (**e_1_**–**e_3_**), and (**f_1_**–**f_3_**) rotated (111) pole figures and (**g**) standard pole figures. (**h_1_**–**h_3_**), (**i_1_**–**i_3_**), and (**j_1_**–**j_3_**) orientation distribution function (ODF) and (**k**) standard ODF maps at φ_2_ = 0° and 45°, where the cube stands for the 45° rotated cube texture.

The original (111) pole figures at x = −3.0, 0.0, and 2.0, after grain refinement occurred through dynamic recrystallization in the pure Al at 0.56*T_m_* and 0.67*T_m_*, are shown in Figure 8a_1_–a_3_,b_1_–b_3_, respectively, whereas those in the high-purity Al at 0.50*T_m_* are depicted in Figure 8c_1_–c_3_. The welding direction (WD)-transverse direction (TD) plane is not the shear plane, which is generally aligned with the surface of a truncated cone with a diameter close to the tool shoulder diameter [12]. To identify the shear texture, the original pole figures were rotated by several angles on the shear direction (SD) and the shear plane normal (SPN) [32]. Figure 8d_1_–d_3_,e_1_–e_3_,f_1_–f_3_ shows the rotated (111) pole figure maps for pure Al at 0.56*T_m_* and 0.67*T_m_*, and the high-purity Al at 0.50*T_m_*, respectively. The orientation distribution function (ODF) maps at φ_2_ = 0° and 45° for the pure Al at 0.56*T_m_* and 0.67*T_m_*, and the high-purity Al at 0.50*T_m_* are represented in Figure 8h_1_–h_3_,i_1_–i_3_,j_1_–j_3,_ respectively. In comparison to the standard (111) pole figure (Figure 8g) and ODF map (Figure 8k), pure Al at 0.56*T_m_* primarily exhibited the B/$\bar{B}$ {112}<110> and C{001}<110> simple shear texture components and the 45° rotated cube texture component at x = −3.0. At x = 0.0 and 2.0, within the peak temperature range, the intensities of the B/$\bar{B}$ and C shear textures gradually increased, as shown in Figure 8h_2_,h_3_. It has been reported that the B/$\bar{B}$ shear texture is predominately developed in Al alloys during FSW [11,12,13,15,18,32], as well as deformation in torsion [33], owing to the significant shear strains [33]. Mironov et al. reported the effect of welding temperature on the texture development of 1050 Al during FSW [11]. A C-type shear texture was developed for the microstructure with a lamellar morphology at a low welding temperature (0.45*T_m_*). As the welding temperature increased to 0.77*T_m_*, the C-type shear texture was transformed to the B/$\bar{B}$ type shear texture, owing to the development of equiaxed grains. In this study, the microstructure consisted of a mixture of equiaxed and elongated grains formed through CDRX and GDRX at a low welding temperature (0.56*T_m_*), as shown in Figure 3d,l. This suggests that the C-type shear texture developed due to the formation of elongated grains, which is generally observed at a reduced welding temperature during FSW [11]. Moreover, the weak 45° rotated cube texture was developed across the entire x-position range. DDRX generally facilitates the development of the 45° rotated cube texture in FCC metals [19,34]. Thus, this result justifies the occurrence of DDRX via HAB bulging, as shown in Figure 5a,b. In contrast, in pure Al at a higher welding temperature of 0.67*T_m_*, only the B/$\bar{B}$-type shear texture became highly evolved across the entire x-position range, as shown in Figure 8i_1_–i_3_. The increase in the welding temperature resulted in the formation of more equiaxed grains through CDRX, leading to the development of a B/$\bar{B}$-type shear texture. The 45° rotated cube component was not observed in this case, as no DDRX occurred during FSW. Based on these results, the resultant texture is related to recrystallization mechanisms including CDRX, GDRX, and DDRX.

In the case of high-purity Al at 0.50*T_m_*, the dominant shear texture was the B/$\bar{B}$ component regardless of the x-position, as shown in Figure 8j_1_–j_3_. The C-type shear texture was not observed, even at the low welding temperature. As shown in Figure 7, the microstructure consisted of equiaxed grains formed through CDRX as a result of the promotion of dynamic recovery induced by the high purification of Al. Therefore, only the B/$\bar{B}$-type shear texture was dominantly developed as in the case of pure Al at a higher welding temperature of 0.67*T_m_*.

## 4. Conclusions

The effects of the welding temperature and Al purity on the microstructural evolution during FSW were examined. Rapid cooling FSW using liquid CO_2_ was applied to reveal the grain refinement mechanism along the material flow path by simultaneously combining the marker insert and tool stop action methods. The following conclusions were drawn:

(1) For the 2N grade pure Al at a low welding temperature of 0.56*T_m_,* DDRX via HAB bulging frequently occurred, particularly in the initial deformation region, leading to the formation of fine grains along the original grain boundaries. As the strain significantly increased, CDRX occurred through the formation of a large number of subgrains within the larger original grains, followed by the transformation from LABs to HABs. In addition, GDRX contributed to the grain refinement;
(2) For the 2N grade pure Al at a high welding temperature of 0.67*T_m_*, the DDRX was suppressed by the promotion of dynamic recovery as a result of the increase in the welding temperature. The more equiaxed and larger grains were formed through the conventional CDRX;
(3) An increase in the Al purity promoted dynamic restoration, leading to acceleration of CDRX and inhibition of DDRX, even at a low welding temperature of 0.50*T_m_* in the 4N grade high-purity Al;
(4) Within the peak temperature range, a fine microstructure was maintained, owing to the dynamic balance between grain refinement through dynamic recrystallization and grain growth by heating effect across all specimens;
(5) The C-type shear texture tended to be developed in pure Al at 0.56*T_m_* with the microstructure consisting of elongated grains formed through GDRX. In addition, the 45° rotated cube texture was developed, as a result of DDRX. In the case where a fine microstructure consisting of equiaxed grains was formed through CDRX, owing to the increase in the welding temperature and high Al purity, the B/$\bar{B}$-type shear texture was highly strengthened in pure Al at 0.67*T_m_* and high-purity Al at 0.50*T_m_*.

## Figures and Tables

**Table 1:** Chemical compositions for pure and high-purity Al (wt%).

Purity	Al	Fe	Si	Cu	Zn	Mn	Mg	Ti	V
2N	Balance	0.31	0.07	0.01	0.01	0.01	0.01	0.02	0.02
4N	Balance	0.001	0.001	0.0015	0.001	–	–	–	–

**Table 2:** Welding conditions and final average grain sizes for pure and high-purity Al, where *T_m_* denotes the melting point (933K) [26].

	Pure Al	High-Purity Al
Peak temp. (K), *T_800_*	523	
Peak temp. (K), *T_1500_*	629	465
*T_800_*/*T_m_*	0.56	
*T_1500_*/*T_m_*	0.67	0.50
Final average grain size (μm) at *T_800_*	2.0	
Final average grain size (μm) at *T_1500_*	4.7	4.8
